# Pathological angiogenesis and inflammation in tissues

**DOI:** 10.1007/s12272-020-01287-2

**Published:** 2020-11-23

**Authors:** Ji-Hak Jeong, Uttam Ojha, You Mie Lee

**Affiliations:** 1 College of Pharmacy, Vessel-Organ Interaction Research Center (VOICE, MRC), Kyungpook National University, Daegu, 41566 Republic of Korea; 2 College of Pharmacy, Research Institute of Pharmaceutical Sciences, Kyungpook National University, Daegu, 41566 Republic of Korea

**Keywords:** Vascular endothelial cells, Angiogenesis, Vascular abnormality, Inflammation, Vascular normalization, Anti-angiogenic therapy, Organ diseases

## Abstract

The role of angiogenesis in the growth of organs and tumors is widely recognized. Vascular–organ interaction is a key mechanism and a concept that enables an understanding of all biological phenomena and normal physiology that is essential for human survival under pathological conditions. Recently, vascular endothelial cells have been classified as a type of innate immune cells that are dependent on the pathological situations. Moreover, inflammatory cytokines and signaling regulators activated upon exposure to infection or various stresses play crucial roles in the pathological function of parenchymal cells, peripheral immune cells, stromal cells, and cancer cells in tissues. Therefore, vascular–organ interactions as a vascular microenvironment or tissue microenvironment under physiological and pathological conditions are gaining popularity as an interesting research topic. Here, we review vascular contribution as a major factor in microenvironment homeostasis in the pathogenesis of normal as well as cancerous tissues. Furthermore, we suggest that the normalization strategy of pathological angiogenesis could be a promising therapeutic target for various diseases, including cancer.

## Introduction

Endothelial cells (ECs) that line the capillary blood vessels in the tissues form numerous networks in the body. Most of the vascular growth is mediated by angiogenesis that is involved in EC proliferation, migration and maturation (Eelen et al. 2018). The formation of vasculature is optimized by the subsequent remodeling and comprises arteries, veins, and capillary beds. Moreover, the vascular network has a well-established function of delivering oxygen, nutrients, hormones, metabolites, and cells as well as regulation of the coagulation processes. In addition to the conventional functions, increasing evidence has indicated that they perform other physiological functions, such as gatekeeping of immune responses (Shetty et al. 2018) and active contribution to the growth or maintenance of the homeostasis in tissue repair or regeneration in the surrounding tissue (Rafii et al. 2016). In the development, tissue-derived signals induce angiogenesis, and ECs release growth factors or directly control or contribute to organ morphogenesis (Ramasamy et al. 2015; Zhu et al. 2020). Moreover, tissue repair and regeneration depend on local ECs. This is owing to the specific signaling molecules, known as angiocrine factor, secreted from the tissue-specific ECs. These tissue-specific ECs play a part of stem cell niches and role in tissue regeneration (Gomez-Salinero and Rafii 2018).

When physiological stress or pathological conditions exceed the capacity of the cells to react and disrupt homeostasis, the tissues experience injury. Inflammation is the primary and the most important reaction to tissues and cell damage. Acute inflammation is a critical protective response to insults by pathogens, toxins, and physical stresses. If this protective response becomes chronic, the inflammation is not resolved, and there is a resultant increase in the number of pathologies, including cancer (Taniguchi and Karin 2018). Emphasis on the tissue microenvironment as well as tumor microenvironment has been recently highlighted in the research of therapeutics (Bahrami et al. 2018; Comen et al. 2018; Roma-Rodrigues et al. 2019). Cross talk between tissue microenvironment, such as parenchymal cells and vascular ECs as well as stromal cells and extracellular matrix, plays an important role in organ function and tumor progression (Belli et al. 2018; Binnewies et al. 2018; Hinshaw and Shevde 2019). In particular, during inflammatory processes, the vascular responsibility is to be permeable to allow inflammatory mediators and immune cells infiltrate to the site of injury or stresses (Aguilar-Cazares et al. 2019). In this review, we have focused on the role of pathological angiogenesis in tumors and organ diseases from a specific viewpoint of ECs as a key player of microenvironment. In addition, the therapeutics by the control of vascular abnormality are also discussed.

## Tumor angiogenesis and inflammation

### Pathophysiological characteristics of tumor endothelial cells and tumor cells under hypoxia

Tumor blood vessels comprise tumor endothelial cells (TECs) or tumor-associated endothelial cells and pericytes (Maishi et al. 2019). TECs line the insides of the vessels, and pericytes surround the vessels externally. These vessels control the passage of oxygen and nutrients and supply them to the tumor cells. Tumor blood vessels are abnormal and differ from normal blood vessels in their morphology, gene expression, and functionality (Maishi et al. 2019; Lugano et al. 2020). The normal endothelial cells (NECs) in normal tissues form a continuous monolayer and tight junctions without overlapping at the margins, while TECs form a defective monolayer and lose their normal barrier function via excessive branching and sprouting. The abnormality of tumor vessels may be caused by defects in pericytes that are loosely attached to TECs and have abnormal shapes (Hashizume et al. 2000; Annan et al. 2020). This abnormality induces intercellular gaps or holes that leak fluid and blood, ultimately resulting in weakening of the vessel wall, increasing the risk of hemorrhage and affecting blood flow.

Tumor hypoxia, defined as insufficient concentration of oxygen (O_2_) in tumor tissues, mainly contributes to tumor angiogenesis (Lee et al. 2017). Robust tumor cells in the tumor tissues grow faster than the vasculature; therefore, a local vascular network is unable to supply oxygen (O_2_) to the tumor cells because of the limit of distance between tumor cells and vasculature. Tumor cells form new blood vessels from the existing vasculature to obtain oxygen and nutrients, i.e. angiogenesis, resulting in formation of structurally and functionally abnormal vasculature. The abnormal vasculature decreases efficiencies for oxygen and drug delivery, which can be a target of antiangiogenic agents acting on normalization of tumor vasculature (Jain 2005; Liu et al. 2018; Du et al. 2019). Under the condition of hypoxia, the tumor cells activate and stabilize a transcription factor hypoxia-inducible factor 1 (HIF-1), a heterodimeric transcription factor that comprises an oxygen-regulated α-subunit (HIF-1α) and a constitutively expressed β-subunit (HIF-1β). This transcriptionally regulates a series of hypoxia-inducible genes, including vascular endothelial growth factor (VEGF) (Rattner et al. 2019).

### VEGF/VEGFR signaling of tumor endothelial cells in tumor angiogenesis

Angiogenesis is regulated in a complex manner via a balance between anti-angiogenic and pro-angiogenic factors. VEGF and angiopoietin families secreted by tumor cells induce tumor angiogenesis, while anti-angiogenic factors, including thrombospondin-1 and angiostatin keep the tumor dormant (Katayama et al. 2019). Among the pro-angiogenic factors, VEGF is the principal pro-angiogenic factor that modulates angiogenesis by binding and interacting with VEGF receptors (VEGFRs). The VEGF expression is detected and upregulated in most human tumors, including breast, brain, and cervical tumors and is correlated with poor prognosis (Dudvarski Stankovic et al. 2018; Li et al. 2018; Martinez-Ordonez et al. 2018). The interaction of VEGF and VEGFR resulted in the activation of the VEGF signaling pathway that promotes tumor angiogenesis by inducing proliferation, sprouting, and migration of TECs and by increasing the microvascular permeability (Lakshmikanthan et al. 2018; Melincovici et al. 2018; Peach et al. 2018). Multiple VEGFs including VEGF-A, VEGF-B, VEGF-C, and VEGF-D interact with VEGF receptors, such as VEGFR1, VEGFR2, and VEGFR3. Among them, VEGF-A/VEGFR2 interaction is the most prominent in the VEGF signaling pathway (Song et al. 2018). VEGF-A binding to VEGFR2 induces receptor dimerization and autophosphorylation at specific tyrosine kinase residues, including Tyr1054 and Tyr1059 within the kinase domain of VEGFR2 (Jin et al. 2018; Chen et al. 2020). VEGF-A induces the activation of diverse signaling pathways, including the phospholipase C-γ (PLC-γ)/protein kinase C (PKC) pathway via VEGFR2 Tyr1175 phosphorylation (Simons et al. 2016) and the phosphoinositide 3-kinase (PI3K)/Akt pathway via VEGFR2 Tyr1175/1173 phosphorylation (Wang et al. 2019b). These activated signaling pathways mediated with VEGFR2 modulate the proliferation and migration of TECs, resulting in the facilitation of vasodilation, permeability, and angiogenic processes (Sadremomtaz et al. 2018; Wang et al. 2019a).

### Recruitment of tumor-associated macrophages by tumor cells under hypoxia

The tumor microenvironment (TME) contributes to tumor growth and progression, comprising innate and adaptive immune cells, such as T cells, dendritic cells, and macrophages (Hinshaw and Shevde 2019). The most abundant population of tumor-infiltrating immune cells in the TME are tumor-associated macrophages (TAMs) that play crucial roles in tumor development, growth, and metastasis by secreting various cytokines, growth factors, and inflammatory mediators (Rhee 2016). Macrophages polarized into two subtypes, classical M1 subtype (M1 macrophages) polarized by helper T cell 1 cytokines or alternative M2 subtype (M2 macrophages) polarized by helper T cell 2 cytokines (Rhee 2016; Atri et al. 2018). M1 macrophages possess antitumor activities by producing pro-inflammatory cytokines, such as IL-1β, IL-6, and TNF-α, while M2 macrophages possess pro-tumor activities by producing anti-inflammatory cytokines, such as IL-10, IL-13, and TGF-β. Although TAMs comprise both, M1 and M2 macrophages, TAMs are generally characterized as M2-like macrophages based on their functions, such as the expression of anti-inflammatory cytokines and angiogenic factors within the TME (Jayasingam et al. 2019).

TAMs that originate from circulating monocytes are recruited to the hypoxic tumor area by tumor-derived factors, such as colony-stimulating factor-1 (CSF-1), VEGF-A, and chemokine (C–C motif) ligand 2 (CCL2) (Lin et al. 2019; Pathria et al. 2019). CSF-1 is a chemotactic factor for most populations of macrophages that induces proliferation, chemotaxis, and differentiation of monocytes (Hua et al. 2019; Pathria et al. 2019). The binding of CCL2 and its receptor, chemokine (C–C motif) receptor 2 (CCR2), mediates the migration and infiltration of monocytes/macrophages to primary or metastatic tumors (Yoshimura 2018; Liu et al. 2020). In addition, CCL2 also binds another receptor CCR4, chemokine (C–C motif) receptor 4, expressed on cytotoxic T lymphocytes, which results in regulating the migration and infiltration of T regulatory cells to tumor sites (Zhang et al. 2006; Nishikawa and Sakaguchi 2010). VEGF-A also recruits and activates monocytes that then differentiate into TAMs in the presence of IL-4 in a xenograft model (Linde et al. 2012). The TAMs also stabilize and constitutively express HIF-1α under hypoxic conditions that facilitates the recruitment of macrophages to hypoxic regions of the tumor via the secretion of chemokines, such as CCL2 and endothelins (ETs) (Tariq et al. 2017; Lin et al. 2019; Lugano et al. 2020). CCL2 secreted by TAMs also contributes to the recruitment of monocytes/macrophages to the hypoxic tumor area, as mentioned above. ETs are small vasoactive peptides that comprise three isoforms (ET-1, ET-2, and ET-3) produced by various types of cells, including macrophages and fibroblasts (Dhaun and Webb 2019). ETs bind to G‐protein‐linked transmembrane receptors, ET-A receptors (ET-RA), and ET-B receptors (ET-RB) (Vercauteren et al. 2017; Dhaun and Webb 2019). ET-2 functions as a chemoattractant for macrophages and THP-1 monocytic cells similar to the CXC chemokines, and the chemoattractant activity is modulated by the ET‐RB receptor (Claudino et al. 2017; Hamilton and Rath 2017).

### Regulation of tumor angiogenesis by tumor-associated macrophages under hypoxia

Based on genetic analysis, TAMs can produce various molecules, including VEGF, TNF-α, IL-1β, IL-8, platelet-derived growth factor (PDGF), thymidine phosphorylase, and MMPs that are involved in tumor angiogenesis (Qian and Pollard 2010; Goswami et al. 2017; Fu et al. 2020). TAMs promote direct tumor angiogenesis by releasing pro-angiogenic factors, such as VEGF in hypoxic areas of the tumors (Lugano et al. 2020). These factors play a role in tumor angiogenesis, similar to that derived from tumor cells. Moreover, TAMs are able to contribute to tumor angiogenesis indirectly via the release of cytokines, such as IL-8 that activate pro-angiogenic signaling pathways in TECs. For instance, IL-8 induced by TAMs interacts with its receptors, CXCR1 and CXCR2, and activates the anti-apoptotic signaling pathway in TECs (Huang et al. 2020). IL-8 signaling either enhances proliferation and survival of TECs or promotes their migration, invasion, and capillary tube organization via the expression of MMP-2 and MMP-9 (Alfaro et al. 2017; Ju et al. 2017). TIE2-expressing macrophages (TEMs) are required for the formation of tumor blood vessels (De Palma et al. 2005; Du Cheyne et al. 2020) and the promotion of tumor angiogenesis (Mazzieri et al. 2011). Tumor hypoxia up-regulates TIE2 receptor expression on TEMs and the production of angiopoietin-2 (ANG-2) in TECs (Murdoch et al. 2008). ANG-2 may recruit TIE2-expressing monocytes to tumors and sites of inflammation, and ANG-2 binds with TIE2, amplifying the production of pro-angiogenic factors. The ANG-2/TIE2 axis, mediates cell-to-cell interactions between TEMs and TECs; further, it initiates angiogenesis by destabilizing existing blood vessels (Lewis et al. 2007; Yan et al. 2017).

### Inflammatory responses by tumor endothelial cells in the hypoxic tumor

As described, various inflammatory mediators secreted by TAMs act directly or indirectly on TECs during the process of tumor angiogenesis. Alternatively, TECs upregulate the production of pro-inflammatory cytokines and chemokines in response to hypoxia that modulates the activities of immune/inflammatory cells and recruit them to the inflammation site. These inflammatory molecules include IL-1β, IL-6, IL-8, CCL2, and TNF-α (Kammerer et al. 2020). As mentioned above, the CCL2 expression is correlated with TAMs recruitment to the tumor and subsequent tumor progression, with high expression resulting in high TAMs accumulation (Choi and Moon 2018; Yang et al. 2019). For instance, the treatment of a neutralizing antibody to CCL2 resulted in a significant reduction in macrophage infiltration, reduced microvessel density, and tumor growth in mouse models of breast cancer (Fujimoto et al. 2009). However, a major source of CCL2 may not be cancer cells or TECs but stroma in these models because there was no significant correlation between the number of TAMs and mRNA levels of human CCL2 in MDA-MB-231 breast cancer cells; however, there was a significant correlation between the number of TAMs and mouse CCL2 in the stroma (Fujimoto et al. 2009). These suggest that the major source of pro-inflammatory molecules is not only tumor cells; further, these molecules expressed from non-tumor cells, including TECs and stroma, are able to recruit macrophages to tumor tissues under hypoxic conditions.

Furthermore, TECs are capable of expressing various growth factors, including CSF-1, VEGF, and PDGF under tumor hypoxia (Pate et al. 2010). CSF‐1 regulates survival, proliferation, and activation of macrophages via binding with CSF-1 receptor (CSF-1R) that belonged to the type III protein tyrosine kinase receptor family. CSF-1/CSF-1R signaling is important for the differentiation and survival of the mononuclear phagocyte system and macrophages (Stanley and Chitu 2014; Dwyer et al. 2017). With the secretion of CSF-1, tumors are able to recruit CSF-1R-expressing macrophages and promote tumorigenesis by enhancing angiogenesis and metastases, resulting in poor survival in various types of tumor (Nakamichi et al. 2013; Stanley and Chitu 2014). The balance between pro-inflammatory and anti-inflammatory cytokines is believed to determine inflammation progression; therefore, TECs can also express anti-inflammatory cytokines, such as IL-10, IL-13, and TGF-β (Shao et al. 2014). Mechanistically, anti-inflammatory cytokines are able to block the progression of the inflammatory responses initiated by pro-inflammatory cytokines.

**Fig. 1:**
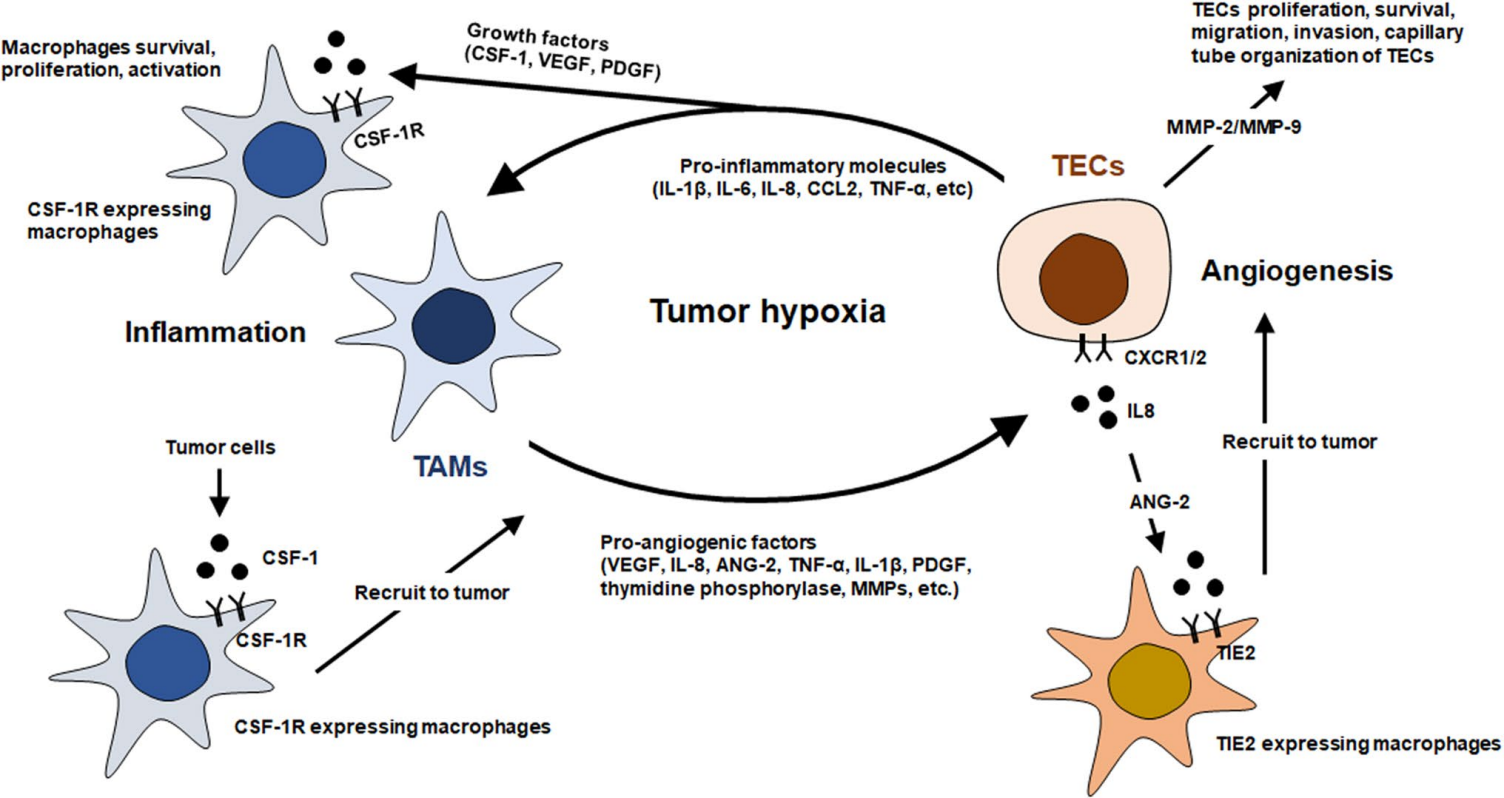
Schematic representation of the interaction between tumor endothelial cells (TECs) and tumor associated macrophages (TAMs) under tumor hypoxia. The inflammation in tumor is primarily associated with the recruitment of tumor associated macrophages (TAMs) by chemotactic factors that are released from tumor cells or tumor endothelial cells (TECs). Thereafter, the recruited TAMs promote tumor angiogenesis by releasing pro-angiogenic factors. Based on the interaction between TECs and TAMs, the relationship between tumor angiogenesis and inflammation form a feed-forward manner

Thus, inflammation in tumor angiogenesis is mainly associated with the recruitment of TAMs originated from the circulating monocytes. During recruitment, chemotaxis is induced by chemotactic factors that are released from tumor cells or TECs. Thereafter, the recruited TAMs promote tumor angiogenesis either directly by releasing pro-angiogenic factors or indirectly by releasing cytokines. Consequently, TECs recruit and activate TAMs under hypoxia, and TAMs enhance TEC proliferation, resulting in the relationship between tumor angiogenesis and inflammation in a feed-forward manner (Fig. 1). Therefore, anti-angiogenic therapies that target tumor inflammation might be a powerful option for cancer treatment.

## Pathological angiogenesis and inflammation beyond tumor

There is increasing evidence showing that angiogenesis and inflammation are linked and play a cooperative role with each other in various inflammatory diseases. These two phenomena assemble together, and hypoxia acts as a common stimulus for both (Costa et al. 2007). Proliferating tissue during inflammation is rich in inflammatory cells, growth factors, macrophage, and other immune cells that release several angiogenic factors under hypoxic conditions (Jackson et al. 1997). In turn, angiogenesis sustains the inflammatory state by supplying the oxygen and nutrients to the inflammatory sites and providing an enormous surface area for the production of necessary cytokines, adhesion molecules, and other inflammatory mediators (Jackson et al. 1997; Costa et al. 2007).

Inflammatory responses affect almost every organ, including the skin, heart, brain, gastrointestinal tract, liver, lung, and kidney, leading to tissue damage or disease. Furthermore, most of them are linked to endothelial dysfunction and pathological angiogenesis (Linlin Chen et al. 2018). Blood vessels are heterogeneous in nature, and ECs not only perform barrier function, but also control the microenvironment in an organ-specific manner (Augustin and Koh 2017). Among the vasculature of different organs, sinusoids of liver show structural and functional diversity having special roles in liver immunity and inflammation (Winkler et al. 2020). Similarly, brain ECs express inflammatory adhesion molecules (E-Selectin and P-Selectin), whereas lung ECs express chemokines (CXCL1 and CXCL9) in response to systemic inflammatory response indicating the distinct organ-specific molecular identity of endothelium (Jambusaria et al. 2020). Therefore, we discuss the role of ECs and pathological inflammation in a few organs.

### Brain

The disorders associated with the vasculature of the brain constitute the second most common cause of death due to circulatory disorders, although the brain vasculature is crucial for development. Angiogenic factor VEGF connects angiogenesis and neurogenesis to the pathogenesis of several neurological disorders and central nervous system (CNS) pathology (Greenberg and Jin 2005). Blood vessels in brain in co-operation with neuronal and perivascular cells form the neurovascular unit, within which the endothelium forms the blood–brain barrier (BBB) (Lee et al. 2020). In contrast to others, CNS ECs form a relatively tight layer, the BBB, because they possess unique properties, such as lack of fenestrae, formation of tight intercellular junctions, slow transcytosis, and creation of a transcellular barrier (Augustin and Koh 2017; Munji et al. 2019). BBB, a vital regulator of the movement of materials between blood and the neural tissue, is not only a wall; it confers several physiological properties and changes in them leads to various CNS disorders (Profaci et al. 2020). Most neurological disorders share the similar changes in EC gene expression profiles during BBB damage and are changed into the cells resembling to the peripheral ECs phenotype (Munji et al. 2019).

**Fig. 2:**
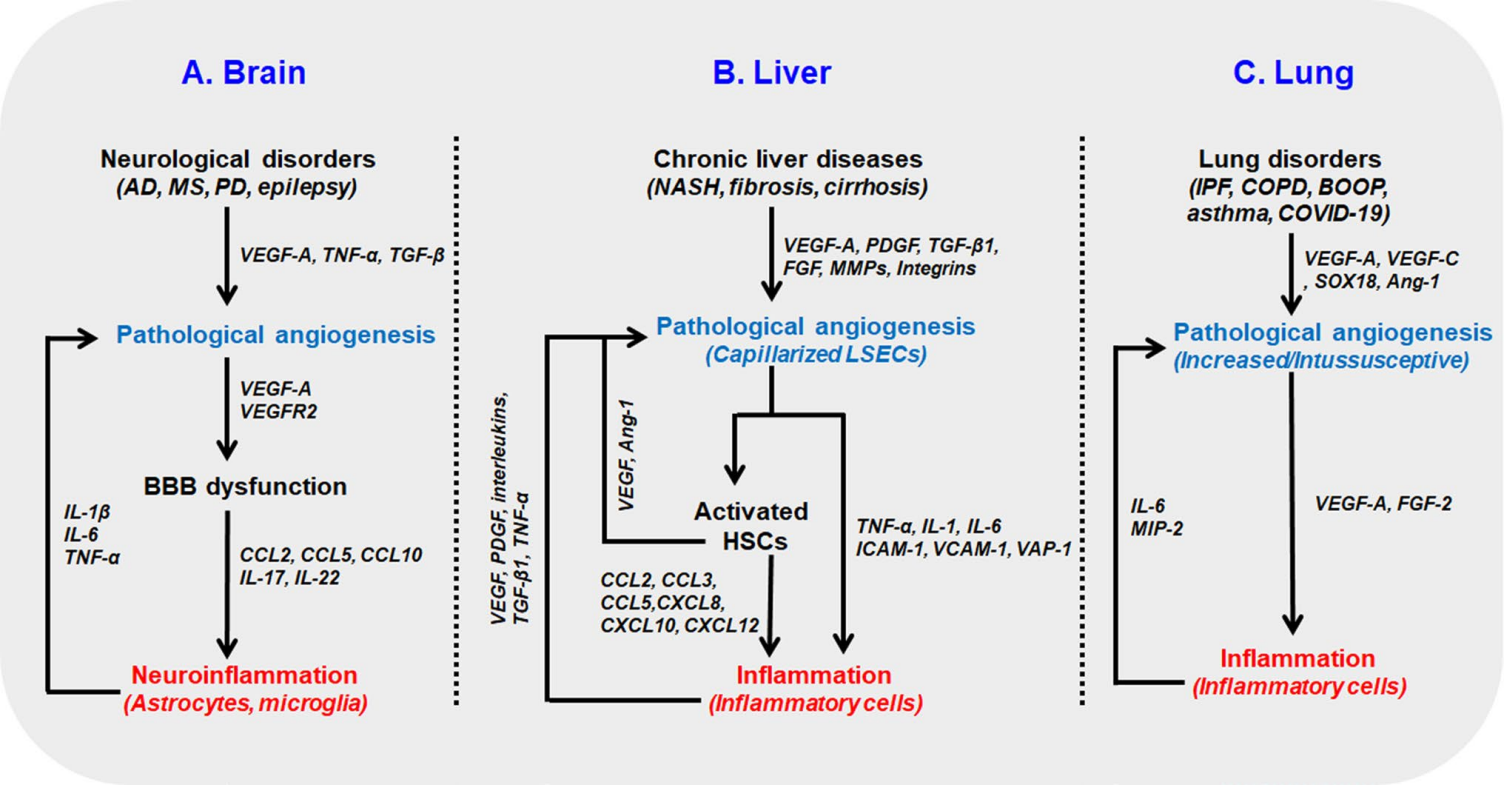
Crosstalk between pathological angiogenesis and inflammation in different tissues **a** In the brain, neurological disorders are characterized by pathological angiogenesis leading to BBB dysfunction through overexpression of angiogenic factors (VEGF-A, VEGFR2). Compromised BBB regulates the expression of proinflammatory chemokines (CCL2, CCL5, and CCL10) and proinflammatory mediators (IL-17 and IL-22) which cause neuroinflammation. In turn, astrocytic cytokines (IL-1β, IL-6, and TNF-α) promote angiogenesis. **b** In liver, the chronic liver diseases are characterized by pathological angiogenesis with capillarized sinusoids which activate hepatic stellate cells (HSCs). Activated HSCs secrete chemokines ligands (CCL2, CCL3, CCL5, CXCL8, CXCL10, CXCL12) and capillarized LSECs secrete cytokines (TNF-α, IL-1, IL-6) and upregulate the cell adhesion molecules (ICAM-1, VCAM-1, VAP-1) leading to inflammation. In turn, inflammatory cells in the damaged liver microenvironment secrete angiogenic factors (VEGF, PDGF, interleukins, TGF-β1, TNF-α) and promote angiogenesis. **c** In lung, pathological angiogenesis can be in the form of increased angiogenesis (IPF, asthma, COPD) or in the form of intussusceptive angiogenesis (COVID-19). Angiogenesis modulator VEGF and FGF-2 cause the remodulation and inflammation of the bronchial cells. In turn, IL-6 and MIP-2 can regulate the inflammation induced angiogenesis in the lung. AD: Alzheimer’s disease, MS: Multiple sclerosis, PD: Parkinson’s disease, VEGF: vascular endothelial growth factor, TNF-α: tumor necrosis factor α, Ang-1: angiopoietin-1, TGF-β: transforming growth factor β, MIP-2: macrophage inflammatory protein 2, IL: interleukin, FGF: fibroblast growth factor, VEGFR: vascular endothelial growth factor receptor, MMP: matrix metalloproteinase, ICAM-1: intracellular adhesion molecule 1, VCAM-1: vascular cell adhesion molecule 1, VAP-1: vascular adhesion protein 1, LSEC: liver sinusoidal endothelial cell, NASH: non-alcoholic steatohepatitis, PDGF: platelet-derived growth factor

Neuroinflammation is the common feature of various neurological disorders, including multiple sclerosis (MS), stroke, Alzheimer’s disease (AD), and Parkinson’s disease (PD) (Gilhus and Deuschl 2019). Compromised BBB in neurological disorders regulate the expression of molecules, such as proinflammatory chemokines (CCL2, CCL5 and CXCL10), proinflammatory mediators (IL-17, IL-22) and movements of ions and cells (microglia and other immune cells, astrocytes), needed for neuroinflammation linking angiogenesis and inflammation in brain disorders (Daneman and Prat 2015) (Fig. 2a). There is strong evidence showing that pathological angiogenesis contributes to AD. Microvascular density is higher in AD patients, and the neuro-selective peptide toxin secreted by ECs leads to neuronal death. Higher level of angiogenic factors VEGF, TNFα, and TGFβ, and the anti-angiogenic properties of drugs effective for AD further strengthens this hypothesis (Vagnucci and Li 2003). Moreover, increased microvascular density determined with CD105 staining and abnormal cerebrovascular tight junction morphology (punctate staining of occludin and zonula occludens-1) were found in Tg2576 mice model of AD (Biron et al. 2011). Similarly, the contribution of angiogenesis in MS pathogenesis, a chronic inflammatory demyelinating disease of CNS, is widely accepted. In guinea pigs with chronic-progressive experimental allergic encephalomyelitis (CP-EAE), an animal model of MS, increased VEGF expression and increased number of blood vessels (factor VIII staining) was found in the infiltrated and demyelinated area (Kirk and Karlik 2003). Immune cells bind to the BBB via adhesion molecules (E-selectin, VCAM-1, ICAM-1) and infiltrate the perivascular space and the parenchyma, causing the release of inflammatory mediators (VEGF, HIF-1, NO, ET-1) from various cells, including astrocytes and microglia that stimulate angiogenic signals in MS (Kirk et al. 2004).

A similar trend has been observed in PD, a neurodegenerative movement disorder characterized by dopaminergic neuron loss and neuroinflammation. PD patients showed an increased level of VEGF, PIGF, and VEGFR2 in the CSF that correlated with more permeable BBB (increased CSF/plasma albumin ratio) and increased neuroinflammation as determined with monocyte chemotactic protein-1 (a marker for glial activation) immunostaining (Shorena Janelidze et al. 2015). Furthermore, increased VEGF levels in the CSF of PD patients has been found to be significantly correlated with BBB dysfunction and neurodegeneration (Zou et al. 2017). Similarly, it is also true that barrier leakage at BBB aggravates epileptic progression and vice versa (Ogaki et al. 2020). During BBB dysfunction, increased VEGF upregulates the expression of matrix metalloproteinase (MMP) in endothelial cells, which activates the VEGF/VEGFR signaling causing excessive angiogenesis in the epileptic brain (Ogaki et al. 2020).

Proinflammatory cytokines (IL-1β, IL-6, and TNF-α) promote the proliferation of endothelial cells, enhancing the angiogenesis during neuroinflammation (Chen et al. 2013). Astrocytic cytokines promote not only inflammation but also angiogenesis AD. In contrast, increased VEGFA in the brain stimulates BBB leakage, secretion of proinflammatory cytokines (MIP-1α), and infiltration of leucocytes causing neuroinflammation (Chen et al. 2013). Finally, we can conclude that most neurological disorders are characterized by pathological angiogenesis coupled with neuroinflammation (Fig. 2a).

### Liver

The liver endothelium is primarily composed of liver sinusoidal endothelial cells (LSECs) located at the interface between adipose tissue derived blood and gut in the one side and hepatocytes and hepatic stellate cells in the other side (Hammoutene and Rautou 2019). Under normal conditions, LSECs are differentiated as having unique structures referred to as fenestrae that are organized in clusters forming sieve plates and lack the basement membrane. Differentiated LSECs function as selective barriers, maintain low portal pressure, regulate sinusoidal blood flow, and maintain the hepatic stellate cells in their quiescent state in normal physiological conditions (Poisson et al. 2017). However, in pathological conditions, they become capillarized (loss of fenestration), promote angiogenesis, and are unable to maintain the hepatic stellate cells in a quiescent state that contributes to fibrosis, inflammation and other chronic liver diseases (DeLeve 2015; Poisson et al. 2017) (Fig. 2b).

Angiogenesis and inflammation are the characteristic features of almost every chronic liver disease. During pathological angiogenesis in the liver, blood vessels are capillarized and aberrantly organized, disturbing the interaction between the ECs and parenchymal cells that activate the immune responses leading to inflammation and chronic liver diseases (Sanz-Cameno et al. 2010). Activated LSECs become proinflammatory and secrete various cytokines and chemokines (TNF-α, IL-1, IL-6 and CCL2) activating the Kupffer cells (Lafoz et al. 2020). Furthermore, during pathological conditions, capillarized and altered LSECs upregulate the cellular adhesion molecules ICAM-1, VCAM-1, and VAP-1, recruiting the blood leucocytes (Lafoz et al. 2020) (Fig. 2B). Hepatic neo-angiogenesis is unique in that it involves the activated hepatic stellate cells, fenestrated sinusoids, and liver-specific angiogenic factor angiopoietin-like 3 (ANGPTL3) (Fernandez et al. 2009). Furthermore, activated stellate cells promote inflammation through secretion of CC chemokines ligands (CCL2, CCL3, and CCL5) and CXC chemokines ligands (CXCL8, CXCL10, and CXCL12) (Zadorozhna et al. 2020) (Fig. 2b).

Inflammatory cells recruited in damaged liver microenvironment secrete angiogenic factors (VEGF, PDGF, PIGF, interleukins, TGF-β1, and TNF-α) to enhance hepatic angiogenesis that further aggravate the inflammation by supplying the metabolic needs, making a loop between the hepatic angiogenesis and inflammation (Coulon et al. 2011). Angiogenesis in chronic liver diseases results from the over-expression of growth factors (VEGF, PDGF, TGF-β1, and FGF), MMPs, integrins αvβ3 and αvβ5, and tissue hypoxia. Hypoxia inducible factor-1α (HIF-1α) upregulates VEGF and angiopoietin-1 (Ang-1) in activated hepatic stellate cells, further aggravating angiogenesis during fibrosis (Fernandez et al. 2009). Ang-2 is responsible for vascular remodeling via upregulation of the expression of endothelial VECAM-1 and ICAM-1, leading to inflammatory cell infiltration. In addition, Ang-2 was elevated in the serum of NASH patients and murine NASH models. Blocking of the ANG-2 pathway ameliorated endothelial dysfunction, steatohepatitis, and liver fibrosis (Lefere et al. 2019). Similarly, inflammatory macrophages are involved in pathological angiogenesis during liver injury and fibrosis (Ehling et al. 2014). These facts sufficiently prove that anti-angiogenic therapies can alleviate liver inflammation and are helpful in chronic liver diseases (Table 1).

**Table 1:** Summary of vascular normalization therapeutics

Drugs/therapies	Mechanism of action	Cell lines or target disease	Limitations	References
VEGF antibody (Bevacizumab)	Neutralization of VEGF	Colorectal cancer and many solid cancers	No significant clinical benefit with monotherapy. Recur tumor hypoxia. Normalization window determination needed	Carmeliet and Jain (2011), Giantonio et al. (2007)
Oxygen microbubbles	Tissue oxygenation	Transgenic prostate adenocarcinoma mouse model	Not effective in tumor diameter over 8 mm	Ho et al. (2019)
VEGFR2 antibody (Ramucirumab for human, DC101 for rat)	Neutralization of VEGFR2	Advanced breast cancer, gastroesophgeal junction carcinoma, hepatocellular carcinoma, metastatic colorectal cancer, non small cell lung cancer	Hypertension	Aprile et al. (2014), Spratlin et al. (2010), Shigeta et al. (2020), Tong et al. (2004), Chauhan et al. (2012)
Everolimus	Inhibition of mTORC1 and HIF-1	Gastric cancer, hepatocellular carcinoma, lymphoma, advanced breast cancer	Patient genome dependence	Atkins et al. (2004)
ABTAA	TIE-2 activation and ANG-2 neutralization	Mouse glioma, breast and lung cancers	No clinical study	Park et al. (2016)
COMP-ANG-1	ANG1 analog TIE-2 activation	Retina vessels	No clinical study	Hwang et al. (2009)
DAAP	Targeting both ANG-2 and VEGF	Mouse ovarian cancer	No clinical study	Koh et al. (2010)

### Lung

The lung vasculature comprises a fine layer of capillary ECs beneath the large surface of alveolar epithelial cells. Angiogenesis and angiogenic factors play a pivotal role in normal lung physiology and development because VEGF is crucial for proper blood vessel formation and normal alveolar development (Thebaud 2007). However, like that in other organs, pathological angiogenesis contributes to several interstitial lung diseases. Idiopathic pulmonary fibrosis (IPF) is linked with angiogenesis because research has shown the presence of neutrophil-activating peptide-78 in IPF patients and increased angiogenesis in bleomycin-induced IPF in rats (Cui et al. 2010). Angiogenic growth factors VEGF-A, VEGF-C, bFGF, and placental growth factor (PLGF) have been found to be increased in cystic fibrosis patients (Eldridge and Wagner 2019). Moreover, SRY-related HMG-box 18 (SOX18), a transcriptional regulator of angiogenesis, is involved in asthma exacerbation (Hong et al. 2020). Moreover, increased VEGF, its receptors, and Ang-1 were found elevated in bronchoalveolar lavage fluid of asthmatic subjects (Eldridge and Wagner 2019). More recently, EC injury, damaged endothelium, abnormal (intussusceptive) angiogenesis, and enhanced sprouting angiogenesis in the lungs have been closely associated with coronavirus disease 2019 (Ackermann et al. 2020).

VEGF and basic fibroblast growth factor (FGF-2), strong modulators of angiogenesis, cause the remodulation and inflammation of bronchial cells which causes several lung disorders like chronic obstructive pulmonary disease (COPD), pulmonary hypertension, asthma and IPF (Laddha and Kulkarni 2019) (Fig. 2c). Furthermore, VEGF upregulated mucin 5AC (Muc5AC) in primary bronchial epithelial cells, revealing the role in hypersecretion of mucus and inflammation in asthmatic patients (Kim et al. 2019).

Inflammation in the lung contributes to asthma, bronchiolitis obliterans organizing pneumonia (BOOP), pulmonary fibrosis, and other disorders. Inflammation-induced angiogenesis also plays a pivotal role in lung diseases. IL-6 and macrophage inflammatory protein-2 (MIP-2) are found to upregulate neo-vascularization in a left pulmonary artery ligation (LPAL) mouse model (Wagner et al. 2008) (Fig. 2c). One study showed VEGF-induced inflammation in a nitric oxide-dependent manner in murine lung (Bhandari et al. 2006). COPD, an inflammatory lung disease, is also characterized by increased angiogenesis with higher VEGF levels in the pulmonary muscular arteries.

### Angiogenesis and inflammation in other tissues

Angiogenesis is the hallmark of numerous inflammatory diseases of not only the liver, brain, and lung, but also of other organs, such as the kidney, skin, gastrointestinal tract, and joints. Normal level of VEGF-A, Ang-1 and vasohibin 1 are essential for the appropriate maintenance of capillaries in kidney and altered expression of proangiogenic and antiangiogenic factors can cause chronic kidney diseases including diabetic kidney disease (DKD and renal fibrosis (Tanabe et al. 2020). In psoriasis, a chronic inflammatory disease of the skin and joints, angiogenic factors and cytokines, such as VEGF, HIF-1α, angiopoietins, TNF-α, and IL-8 are elevated (Heidenreich et al. 2009). Similarly, increased angiogenesis with higher level of VEGF-A and Ang-2 is closely associated with sepsis, another skin inflammatory disorder (Faiotto et al. 2017). In rheumatoid arthritis (RA), an autoimmune inflammatory disorder, angiogenesis promotes the infiltration of inflammatory cells into the joints, resulting in damage to the synovial tissue. In turn, fibroblasts and macrophages from the damaged synovial tissue release cytokines (TNF-α, IL-1β, IL-6, and IL-8) that regulate the expression of adhesion molecules, matrix metalloproteinases, chemokines, and growth factors leading to extensive angiogenesis (Elshabrawy et al. 2015). Microvascular remodeling and extensive angiogenesis play a crucial role in inflammatory bowel disease (IBD), a challenging inflammatory disease of gastrointestinal tract (Pousa et al. 2008). In IBD patients, higher levels of VEGF, endothelial αVβ3, TNF-α, and bFGF were found along with increased vessel density (Danese et al. 2006). Another example of pathological angiogenesis is diabetic retinopathy, a major cause of visual loss and blindness globally (Wang et al. 2012). Capillary occlusion leads to the hypoxia that in turn stimulates growth factors (VEGF, IGF-1, Ang-2), integrins (αvβ3, αvβ5) and metalloproteases (MMP-2, MMP-9, urokinase), causing endothelial cell migration and proliferation in DR (Simó 2007). Hyperglycemia leads to nitric oxide synthase dysregulation and the formation of reactive oxygen species (ROS) and glycation end products that activate the NF-κB pathway causing EC activation and infiltration of inflammatory cells, linking angiogenesis and inflammation in DR patients (Zhang et al. 2011).

### Therapeutics strategy for pathological angiogenesis by normalization

The importance of angiogenesis in chronic inflammation has led to the application of anti-angiogenic strategies to treat inflammatory diseases in the organ and tumor. Such approaches include blocking the VEGF function or receptor activation with novel agents. The first clinical trial for anti-angiogenic therapy in human solid tumor was performed with anti-VEGF antibody, bevacizumab; however, significant clinical benefit was not observed with its monotherapy (Giantonio et al. 2007; Carmeliet and Jain 2011). However, further clinical trials have confirmed that in combination with the standard first-line chemotherapy regimens significantly improved patient outcomes (Hurwitz et al. 2004; Saltz et al. 2008), suggesting that anti-VEGF therapy increases the efficacy of systemic chemotherapy in some types of cancers. In contrast, further serial clinical data showed discouraging results in that anti-VEGF therapy increases the metastatic potential and chemoresistance (Padera et al. 2008; Welch et al. 2010; Miles et al. 2011), indicating that tumor hypoxia recurs with anti-angiogenic therapy (Pennacchietti et al. 2003). In 2001, Jain et al. (Jain 2001) proposed the “vascular normalization” hypothesis, according to which, optimal anti-angiogenic therapy can normalize the abnormal structure and function of the tumor vasculature without eliminating vessels. During the previous two decades, many trials have demonstrated that vascular normalization improves the delivery and efficacy of drugs and radiotherapy and immune cell infiltration, and reduces the hypoxic region of the tumor (Wu et al. 2018). In contrast to the anti-angiogenic therapy, vascular normalization is to be directed to reduce vascular permeability and interstitial fluid pressure and improve blood flow and perfusion to the tissues without devastating vessel regression (Goel et al. 2012; Fukumura et al. 2018).

Therefore, the first strategy to normalize the pathological angiogenesis is mainly focused on VEGF. In addition to the neutralization of VEGF with its antibody, transcription activators, such as HIF-1 (hypoxia) and STAT-3, and microRNAs that regulate VEGF mRNA stability are promising targets for vascular normalization via the inhibition of VEGF expression (Wu et al. 2018). Small molecules including EZN-2968, Topotecan, PX-478, 2-methoxyestradiol, and KC7F2 that inhibit HIF-1α have been developed because tumor hypoxia stabilizes the HIF-1α engaged in VEGF expression and chemoresistance in many solid tumors (Yu et al. 2017). Many HIF-1α inhibitors are yet to be investigated; however, the vascular normalization when tumor growth is inhibited, and further experimental evidence showing that the small molecule HIF-1α inhibitors induce normalization of tumor vasculatures is required. A recent interesting approach to enhance the local oxygen level in the tissues has used the oxygen microbubble with the ultrasound method. With the ultrasound, oxygen microbubbles improved the morphology and function of the tumor vasculature via increased tumor oxygenation and inhibited HIF-1α and VEGF expression (Ho et al. 2019). This ultrasound perfusion of oxygen gives a feasibility of vascular normalization in cancer treatment and organ diseases in clinics via modulation of the hypoxic region of the tissues.

DC101 is a rat monoclonal antibody against VEGFR2 that is highly expressed in tumor ECs and mediates most of the angiogenic properties of VEGF. Specific blockage of the VEGFR2 pathway by DC101 induced vessel normalization in mice with lung, breast, colorectal, and glioblastoma multiform tumors (Tong et al. 2004; Chauhan et al. 2012). Dual inhibition of VEGFR2 and programmed death receptor-1 (PD-1) with their specific antibodies decreased primary tumor growth via increased vascular normalization and anti-tumor immune responses in hepatocellular carcinoma (Shigeta et al. 2020). Ramucirumab is a humanized monoclonal antibody against VEGFR2 that has been approved for patients with advanced or metastatic breast cancer or gastroesophageal junction carcinoma and other cancers (Aprile et al. 2014). Ramucirumab decreased the vascularity and permeability in a liver metastasis; however, to our knowledge, no study has assessed vascular normalization (Spratlin et al. 2010). Everolimus has been approved from US FDA for the treatment of various cancers as an mTOR inhibitor. This drug exerts significant effects on the decreased expression of HIF-1 and VEGF in several tumor models (Atkins et al. 2004). Bevacizumab and everolimus, in combination with chemotherapy, coordinately target the angiogenic signaling pathway and achieve a synergistic effect to improve vascular normalization and suppress potential chemoresistance (Manegold et al. 2008).

In addition to the VEGF signaling pathway, the TIE2/angiopoietin pathway is an alternative target for normalizing the tumor vessel structure. ANG-2 and TIE2 dual binding antibody, called ABTAA, exerts ANG-2-neutralizing and TIE2-activation properties that enhance vascular normalization in gliomas, Lewis lung carcinoma, and MMTV-PyMT breast cancer models (Park et al. 2016). When ABTAA was combined with temozolamide, the standard chemotherapy for glioma treatment, tumor volume was inhibited by 75% as compared to 39% with ABTAA alone, suggesting that ABTAA enhanced drug delivery. This study also suggested that ABTAA improved the tumor microenvironment via increased infiltration of M1-like macrophages into tumors. Activation of TIE2 in the tumor ECs by an ANG-1 analog, COMP-ANG-1, also increased tight endothelial junctions and pericyte coverage that alleviated hypoxia and enhanced the effect of cytotoxic drugs (Hwang et al. 2009). Strategic targeting of both, ANG-2 and VEGF-A, with a bispecific trap, double anti-angiogenic protein, named DAAP, induced vessel normalization and markedly reduced vessel leakage in an ovarian cancer ascites model (Koh et al. 2010).

It is noteworthy that the use of ABTAA was used for treating sepsis (Han et al. 2016), a disease associated with inflammation, like cancer, vascular leakage, and increased ANG-2 levels (Parikh et al. 2006; Siner et al. 2009). Using a novel murine model, severe sepsis (high-grade cecal ligation and puncture, CLP), and *S. aureus* inoculation or LPS injection, the results showed that treatment of ABTAA reduced cytokine storms and vascular leakage and strengthened the endothelial glycocalyx. ABTAA use significantly reduced organ damage and sepsis mortality in the tested models (Han et al. 2016). These reports suggest that targeting the TIE2/ANG axis causes vascular normalization and reduces inflammation in cancer, sepsis, and various organ diseases. The contribution of angiogenesis in chronic inflammation also prompts the application of established therapies, including thalidomide, sulfasalazine, methotrexate, penicillamine, and anti-TNF reagents to inhibit angiogenesis; this may explain some of the claims for their efficacy in chronic inflammatory diseases (Lai and Adams 2005).

The time window for utilization (i.e., the administration of additional therapies) of the vessel normalization phase after anti-VEGF/VEGFR2 is very narrow. Considering the lengthy chemotherapy or radiotherapy regimens used in the clinic setting, the dose-dependent effect and narrow normalization window involved with VEGF blockade is an active area of research; therefore, other strategies need to be explored. Based on the similarities of the cancer tissues with the pathology of organ diseases with abnormal vasculatures and inflammation, it can be extrapolated from the cancer research to the other organ diseases. Thus, the judicious use of antiangiogenic therapy refines some vessels and changes the grossly abnormal structure and function of the remaining vasculature to a more normal state, preventing its harmful effects on the pathological tissue microenvironment.

## Conclusion

In this review, we discuss the significance of vascular-organ interaction during pathological conditions. At first, we speculated that the interaction between TECs and TAMs highlighted the correlation between angiogenesis and inflammation in tumor. Furthermore, we summarized not only the tumor but also the crosstalk between pathological angiogenesis and inflammation in different tissues with major focus on the brain, liver, and the lung during several organ-specific diseases. Finally, we suggest the normalization strategies for pathological angiogenesis. Thus, this review provides a detailed explanation for pathological angiogenesis in tumor and normal tissues and idea for anti-angiogenic therapies to treat the inflammatory disorders.
